# Demographic Comparison of Information Security Behavior Toward Health Information System Protection: Survey Study

**DOI:** 10.2196/49439

**Published:** 2023-08-24

**Authors:** Puspita Kencana Sari, Putu Wuri Handayani, Achmad Nizar Hidayanto

**Affiliations:** 1 Faculty of Computer Science Universitas Indonesia Depok Indonesia; 2 Faculty of Economics & Business Telkom University Bandung Indonesia

**Keywords:** behavioral research, health information system, human activities, information security, mobile security

## Abstract

**Background:**

The health information system (HIS) functions are getting wider with more diverse users. Information security in the health industry is crucial because it involves comprehensive and strategic information that might harm human life. The human factor is one of the biggest security threats to HIS.

**Objective:**

This study aims to investigate the information security behavior (ISB) of HIS users using a comprehensive assessment scale suited to the information security concerns in health care. Patients are increasingly being asked to submit their own data into HIS systems. As a result, this study examines the security behavior of health workers and patients, as well as their demographic variables.

**Methods:**

We used a quantitative approach using surveys of health workers and patients. We created a research instrument from 4 existing measurement scales to measure prosecurity and antisecurity behavior. We analyzed statistical differences to test the hypotheses, that is, the Kruskal-Wallis test and the Mann-Whitney test. The descriptive analysis was used to determine whether the group exhibited exemplary behavior when processing the survey results. A correlational test using the Spearman correlation coefficient was performed to establish the significance of the relationship between ISB and age as well as level of education.

**Results:**

We analyzed 421 responses from the survey. According to demographic factors, the hypotheses tested for full and partial security behavior reveal substantial differences. Education levels most significantly affect security behavior differences, followed by user type, gender, and age. The health workers’ ISB is higher than that of the patients. Women are more likely than men to engage in prosecurity actions while avoiding antisecurity behaviors. The older the HIS user, the more likely it is that they will participate in prosecurity behavior and the less probable it is that they will engage in antisecurity behavior. According to this study, differences in prosecurity behavior are mostly impacted by education level. Higher education, on the other hand, does not guarantee improved ISB for HIS users. All demographic characteristics, particularly concerning user type, show discrepancies that are caused mainly by antisecurity behavior rather than prosecurity behavior.

**Conclusions:**

Since patients engage in antisecurity behavior more frequently than health workers and may pose security risks, health care facilities should start to consider information security education for patients. More comprehensive research on ISB in health care facilities is required to better understand the patient’s perspective, which is currently understudied.

## Introduction

### Background

The COVID-19 pandemic has progressively pushed health care institutions to use information technology solutions in providing health services that might raise the exposure to information security threats such as phishing emails, ransomware attacks, and network outages [1]. Ransomware attacks are increasingly targeting hospitals in many nations [2], disrupting health care operations and putting patients’ lives in danger. During the COVID-19 pandemic, the work from home policy required organizations, including health care institutions, to activate their information systems so that they could be accessible from outside the facility [3]. Cyberattacks have grown during the COVID-19 pandemic as a result of personnel lacking an adequate level of security knowledge to work from home [4].

The health information system (HIS) functions are getting wider, from the electronic medical record system to the current telemedicine system to provide web-based health services. HIS users, including health workers and patients, are also expanding. Information security has become an essential factor to be evaluated in HIS implementation [5]. The security threat from human factors is increasing because of diverse users. Meanwhile, patients as research participants are rarely studied empirically [6]. Many instances of security breaches in health care providers are brought on by human behavior [7-9]. Therefore, it is significant to understand HIS users’ information security behavior (ISB) to determine the proper information security controls.

Security behavior intentions might be divided into 2 main categories [10]. First, prosecurity behavior refers to the intention to support information security, including complying with security policies and assuring security controls using required tools [11]. Second, antisecurity behavior reflects the intent of disruptive information security, including violating security policies, making risky use of information system resources, and dismissing security requirements [11].

This study answers the research call from Ahouanmenou et al [12] that stated a gap related to information security studies in hospitals where less research has been carried out on how humans affect information security-related incidents. Most of the studies focus on protection technology, processes, and procedures. Advanced information systems require knowledgeable individuals to prevent security breaches following established security requirements, so the information security knowledge and expertise of health professionals must be quantified [13]. Therefore, this study aims to understand the ISB of HIS users based on a complete measurement scale adjusted to the information security risks in health care and linked to demographic differences. Academic contributions to information security research, mainly empirical measurement of HIS users’ ISB. A practical contribution is provided by proposing appropriate training approaches to raise security awareness in the health care organization.

There are several frameworks for measuring ISB from previous literature, namely the Human Aspects of Information Security Questionnaire (HAIS-Q) [14,15], Security Behavior Intentions Scale (SeBIS) [16], Risky Cybersecurity Behaviors Scale (RScB) [17], and counterproductive computer security behaviors (CCSB) [18]. HAIS-Q and SeBIS focus on prosecurity behaviors that support information security protection. In contrast, RScB and CCSB focus on antisecurity behaviors that risk causing information security incidents.

### Previous Work

Previous studies in the health care context mostly used the HAIS-Q [19-22], a study [19] used HAIS-Q and SeBIS, and a study used the RScB [13]. The majority of studies [13,19-21] investigate health workers as research participants, while a study [22] examine patient behavior as an end user of clinical mobile apps. The riskiest behavior of health workers is visiting external websites using the hospital’s computer [22], while patients rarely choose complex passwords and change passwords in their mobile health (mHealth) apps [22]. Demographic factors, such as age, gender, and education level, are commonly used to predict ISB. However, different results were obtained from previous research. Previous studies [23-26] revealed a difference in ISB of health care professionals based on age, while a study [13] showed otherwise. As well as gender, a significant difference in ISB was established in a study [25] but not in other studies [13,23,24]. Meanwhile, education level is still less explored and found significant to ISB in a study [24] but not substantial in another study [23].

### Goal of This Study

This study uses a quantitative method to investigate ISB from 2 types of HIS user, health worker and patient, which are still understudied. We also examine the differences between ISB according to demographic factors. Therefore, this study has 2 research questions (RQ) to be addressed:

- RQ1: Is there any difference in HIS users’ ISB according to user type, age, gender, and education?
- RQ2: How does HIS users’ ISB differ according to user type, age, gender, and education?

This study has 4 hypotheses to address the RQs as follows:

- Hypothesis 1: There are differences in security practices between health workers and patients.
- Hypothesis 2: There are differences in security practices between male and female.
- Hypothesis 3: There are differences in security practices across age categories.
- Hypothesis 4: There are differences in security practices across education categories.

## Methods

### Population and Sampling

The population of this study was users of HIS applications managed by health facilities in Indonesia as a case study context. Indonesia has the lowest score on the cybersecurity index for G20 countries [27]. Most Indonesian health care providers have inadequate information security policies due to a lack of national health information security regulation. Meanwhile, the government is encouraging the HIS implementation in all health care facilities. By understanding the ISB, we may take the first step toward building better health information security policies.

The sampling technique is nonprobability sampling, especially purposive sampling, according to the established criteria:

1. Patients or health workers who have user account in HIS; and
2. HIS is provided and managed by health facilities through both website-based and mobile apps.

### Research Instrument

Table 1 describes each framework’s theoretical background, measurement scope and indicators, and measurement scale. This study establishes a modified ISB measurement scale from 4 ISB frameworks in Table 1 as research instruments. We use a 5-point scale (“never” to “always”) to refer to SeBIS. There are 28 behaviors as research indicators in this study, consisting of 14 prosecurity behaviors and 14 antisecurity behaviors. The mapping of each indicator with the related framework can be seen in Table 2.

In general, we divide the ISB indicators into 4 focus areas, that is, device protection (code 1.1 to 1.6), password management (code 2.1 to 2.6), proactive awareness (code 3.1 to 3.10), and information handling (code 4.1 to 4.6). We collected data through web-based and offline surveys of several health care facilities in Indonesia. We asked for respondents’ consent before they fulfill the questionnaire.

**Table 1:** Information security behavior measurement frameworks.

Framework	Underpinning theory	Scope and indicators	Likert scale
SeBIS^a^	We derived from internet and computer security best practices	16 items from 4 dimensions: (1) device securement, (2) password generation, (3) proactive awareness, and (4) updating behavior	5-point scale (never to always)
HAIS-Q^b^	KAB^c^ model and empirical studies related to human error.	63 items from 3 dimensions (knowledge, attitude, and behavior) with 7 focus areas: (1) password management, (2) email use, (3) internet use, (4) social networking site use, (5) incident reporting, (6) mobile computing, and (7) information handling	5-point scale (strongly agree to strongly disagree)
CCSB^d^	Mostly correlated to social cognitive theory	12 items from 3 dimensions: (1) careless use of IS resources, (2) procrastinating in carrying out required IS actions, and (3) improper use of IS resources	7-point scale (almost never to almost always)
RScB^e^	Partly based on the SeBIS measurement scale	20 items to assess behaviors that may induce poor cybersecurity practices	7-point scale (never to daily)

^a^SeBIS: Security Behavior Intentions Scale.

^b^HAIS-Q: Human Aspects of Information Security Questionnaire.

^c^KAB: Knowledge, Attitude, Behavior.

^d^CCSB: counterproductive computer security behavior.

^e^RScB: Risky Cybersecurity Behaviors Scale.

**Table 2:** Information security behavior (ISB) indicators in research instruments.

Item code	ISB indicators	ISB frameworks			
		SeBIS^a^	HAIS-Q^b^	RScB^c^	CCSB^d^
1.1	Locking workstation when idle	✓	✓		
1.2	Using passwords to unlock devices	✓			
1.3	Physically securing mobile devices		✓		✓
1.4	Not logging out of secure systems after use^e^				✓
1.5	Not checking for software (antivirus, operation system) update^e^	✓		✓	✓
1.6	Disabling the antivirus to download from websites^e^			✓	
2.1	Using strong password	✓	✓	✓	✓
2.2	Using a different password for different account	✓	✓	✓	
2.3	Updating work-related passwords regularly				✓
2.4	Pasting or sticking computer passwords in a visible place^e^				✓
2.5	Password sharing^e^		✓	✓	✓
2.6	Never change the default password^e^	✓	✓		
3.1	Submit information on the internet (after checking certification)	✓	✓	✓	
3.2	Clicking on links (after verifying the source)	✓	✓	✓	
3.3	Opening attachments in emails from a trusted sender		✓		
3.4	Social media privacy setting		✓		
3.5	Accessing dubious or nonrelated websites^e^	✓	✓		✓
3.6	Downloading file (antivirus, digital media, data, and material from unknown source)^e^		✓	✓	✓
3.7	Sending sensitive information through Wi-Fi^e^		✓	✓	
3.8	Sharing sensitive information or posting about work on social media^e^		✓	✓	
3.9	Reporting all incidents		✓		
3.10	Ignoring poor security behavior by colleagues^e^		✓		
4.1	I am disposing of sensitive printouts properly		✓		
4.2	I am never leaving sensitive material		✓		
4.3	Backing up data files as frequently as possible				✓
4.4	Not always treating sensitive data carefully^e^			✓	✓
4.5	Sending personal information to strangers (through instant messaging)^e^			✓	
4.6	Sending personal information to strangers (through a website)^e^			✓	

^a^SeBIS: Security Behavior Intentions Scale.

^b^HAIS-Q: Human Aspects of Information Security Questionnaire.

^c^RScB: Risky Cybersecurity Behaviors Scale.

^d^CCSB: counterproductive computer security behaviors.

^e^antisecurity behavior.

### Data Analysis Technique

A statistical differences analysis of the sample according to users’ type, age, gender, and education will be conducted to analyze survey results. A test of normality (Kolmogorov-Smirnov and Shapiro-Wilk) will be conducted to define whether a parametric or nonparametric test should be performed [28]. When some data fail to meet the normality assumption test (asymptotic significance value *P*<.05), the researcher will use nonparametric statistical procedures such as the Mann-Whitney test for comparing 2 independent samples or the Kruskal-Wallis test for comparing more than 2 independent samples [29]. A descriptive analysis (mean, median, and SD) of the sample in the different variables shows which group has better ISB [30]. The higher mean value indicates better ISB, which means more frequently adopting prosecurity practices and less frequently adopting antisecurity practices. Furthermore, a correlative analysis was carried out to determine the significance of the correlation between ISB and age and level of education, which are ordinal variables. The correlative test uses the Spearman correlation coefficient, with a significance value of (2-tailed) *P*<.05 indicating a significant association between ISB indicators and the age or educational level of HIS users. Meanwhile, the mean value of each group may be used to analyze the significance of the correlation between security behavior, gender, and user type.

### Ethical Approval

Respondents will not be harmed in any way as a result of their participation in this study. The participants provide written informed consent before the trial. The privacy of research respondents will be maintained. Respondents’ voluntary involvement in the study will be regarded as extremely valuable. Respondent data are admissible only for the purpose of this study. Any deceptive details, as well as biased depictions of the main data findings, should be avoided. This study has been reviewed and approved by Manager of Research and Community Services, Faculty of Computer Science, Universitas Indonesia (IRB approval number S-252/UN2.F11.D1.5/PPM.00.00/2023).

## Results

### Demographic Characteristics

**Figure 1:**
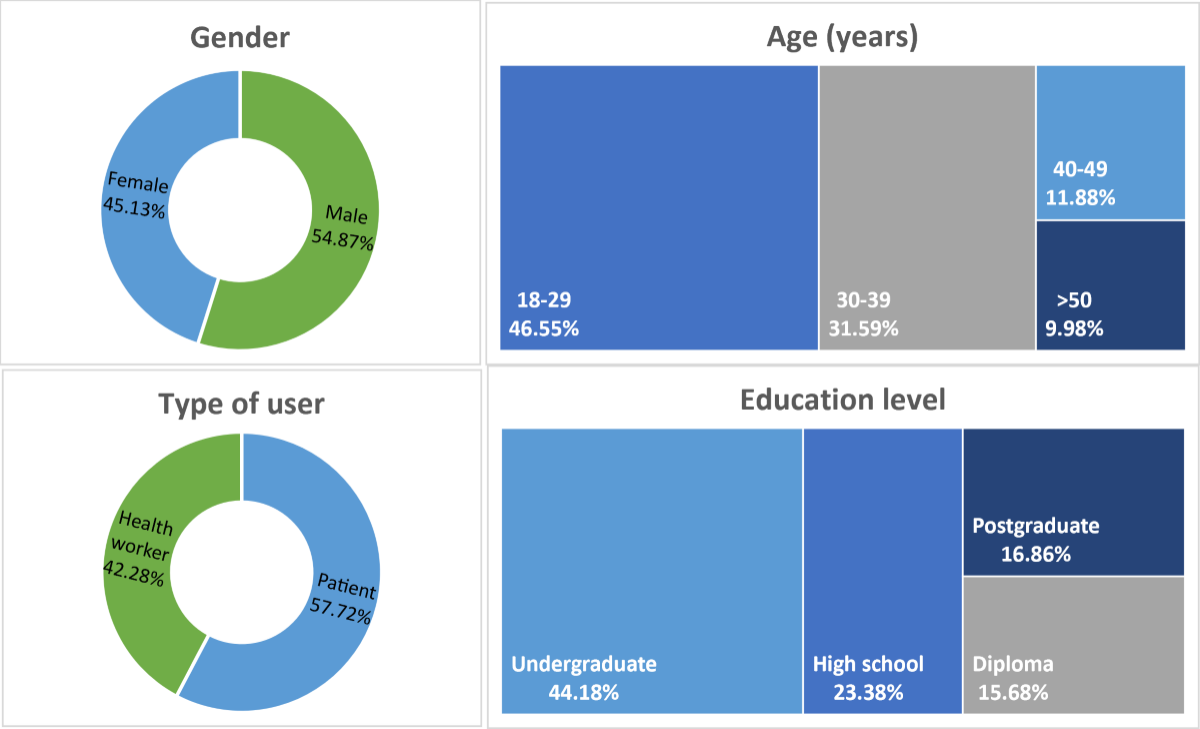
Demographic details of respondents.

We collected 564 responses through the survey from March to May 2022. After validating, 143 responses were excluded since they did not fulfill the respondents’ criteria and were not completed. We processed 421 responses to be analyzed further. Figure 1 shows respondents’ characteristics in this study.

### Validity and Reliability Test

The findings of the research instrument’s validity test with 28 indicators reveal a significance value of *P*<.05, except for indicator 3.5. So, indicator 3.5 was deleted from the instrument and recalculated. Then, for 27 indicators, a revalidity test was performed, and the results are displayed in Table 3. It shows that all items in the instrument are valid. The Cronbach α rating for the research instrument reliability test with 27 indications was .793. Cronbach α values of .70 or above are typically deemed acceptable (Hair et al [31]). This demonstrates that the instrument used is valid and trustworthy for assessing HIS users’ ISB.

**Table 3:** Instrument validity test results.

Item code	Pearson correlation coeeficient	*P* value
1.1	0.432	<.001
1.2	0.470	<.001
1.3	0.493	<.001
1.4	0.371	<.001
1.5	0.300	<.001
1.6	0.202	<.001
2.1	0.472	<.001
2.2	0.404	<.001
2.3	0.284	<.001
2.4	0.378	<.001
2.5	0.408	<.001
2.6	0.227	<.001
3.1	0.372	<.001
3.2	0.590	<.001
3.3	0.290	<.001
3.4	0.471	<.001
3.6	0.421	<.001
3.7	0.344	<.001
3.8	0.430	<.001
3.9	0.514	<.001
3.10	0.264	<.001
4.1	0.580	<.001
4.2	0.474	<.001
4.3	0.460	<.001
4.4	0.377	<.001
4.5	0.366	<.001
4.6	0.463	<.001

### Hypothesis Testing

The reliability test for the research instrument shows Cronbach α value is .785. Cronbach α is commonly regarded as having an acceptable range of .70 or above [31]. The normality test result shows all the data are not normal (*P*<.05); therefore, we use a nonparametric test for the hypothesis testing, namely the Mann-Whitney *U* test and the Kruskal-Wallis test.

All hypotheses are accepted for overall ISB where the hypotheses testing shows significant results according to the user’s type (*P*<.001), gender (*P*<.001), age (*P*=.008), and education (*P*<.001). Table 4 shows the hypothesis testing result (*P* value) for each ISB indicator. Total 7 behaviors (1.2, 1.4, 2.3, 2.4, 3.6, 4.1, and 4.4) are significantly different based on 4 demographic factors, while 3 others (1.1, 2.1, and 2.2) are not significantly different for all factors. The most behavioral differences are based on the education level, followed by user’s type, gender, and age. The differences are dominated by antisecurity behavior in all demographic factors.

**Table 4:** Hypothesis testing result.

Item code	Mann-Whitney test (*P* value)		Kruskal-Wallis test (*P* value)		Conclusion
	User’s type	Gender	Age	Education	
1.1	.86	.89	.13	.22	All hypotheses are rejected
1.2	.007	.01	.04	.002	All hypotheses are accepted
1.3	.11	.68	.23	.03	H4^a^ is accepted
1.4	<.001	<.001	<.001	.001	All hypotheses are accepted
1.5	<.001	.25	.16	.004	H1^b^ and H4 are accepted
1.6	.005	.24	.006	.04	H1, H2^c^, and H4 are accepted
2.1	.43	.29	.13	.41	All hypotheses are rejected
2.2	.25	.31	.30	.11	All hypotheses are rejected
2.3	.001	<.001	<.001	.02	All hypotheses are accepted
2.4	.002	.02	.01	.02	All hypotheses are accepted
2.5	.048	.47	.09	.47	H1 is accepted
2.6	<.001	.63	.34	.08	H1 is accepted
3.1	.69	.84	.24	.02	H4 is accepted
3.2	.68	.049	.09	.82	H2 is accepted
3.3	.37	.77	.45	.01	H4 is accepted
3.4	.78	.66	.002	.17	H3^d^ is accepted
3.6	<.001	<.001	<.001	<.009	All hypotheses are accepted
3.7	.06	.006	.005	.02	H2, H3, and H4 are accepted
3.8	<.001	.002	.07	.28	H1 and H2 are accepted
3.9	<.001	.002	.46	.002	H1, H2, and H4 are accepted
3.10	.04	.007	.02	.81	H1, H2, and H3 are accepted
4.1	.001	<.001	.04	.001	All hypotheses are accepted
4.2	.16	.01	.56	.25	H2 is accepted
4.3	.87	.90	.02	.01	H3 and H4 are accepted
4.4	<.001	<.001	.005	.02	All hypotheses are accepted
4.5	<.001	.001	.21	.001	H1, H2, and H4 are accepted
4.6	.001	<.001	.16	.003	H1, H2, and H4 are accepted

^a^H4: hypothesis 4.

^b^H1: hypothesis 1.

^c^H2: hypothesis 2.

^d^H3: hypothesis 3.

### Descriptive Analysis

Table 5 shows the results of the descriptive analysis calculation for each behavior indicator. The bigger the mean or median value, the better the ISB since it suggests that security behavior is performed more frequently for prosecurity indicators and less frequently for antisecurity indicators. There are some information security practices that still need to be improved based on the mean and median values in Table 5. First, in terms of device security, more respondents do not use the device screen lock feature to get access to the HIS with a password (item 1.1). Furthermore, respondents almost never signed out after using HIS (item 1.4) and upgrading critical apps such as antivirus and operating systems (item 1.5). Second, in terms of password management, many respondents still do not update their passwords on a regular basis (item 2.3). Third, when it comes to proactive awareness in using the internet, social media, email, and incident reporting, many respondents continue to provide information to websites without first confirming their legitimacy and security (item 3.1). Furthermore, respondents continue to allow colleagues or acquaintances to violate information security (item 3.10).

**Table 5:** Descriptive statistics.

Item code	Mean (SD)	Median
1.1	3.15 (1.47)	3
1.2	3.88 (1.38)	5
1.3	3.66 (1.28)	4
1.4	3.25 (1.47)	3
1.5	3.29 (1.16)	3
1.6	3.85 (1.32)	4
2.1	4.01 (1.27)	5
2.2	3.48 (1.38)	4
2.3	2.46 (1.23)	2
2.4	4.38 (1.10)	5
2.5	4.42 (1.02)	5
2.6	3.56 (1.46)	4
3.1	3.25 (1.33)	3
3.2	4.35 (0.97)	5
3.3	3.69 (1.28)	4
3.4	3.53 (1.18)	4
3.6	3.80 (1.07)	4
3.7	3.78 (1.14)	4
3.8	4.47 (0.90)	5
3.9	3.61 (1.31)	4
3.10	3.43 (1.15)	4
4.1	3.85 (1.29)	4
4.2	4.00 (1.28)	4
4.3	3.55 (1.30)	4
4.4	4.02 (1.15)	4
4.5	4.26 (1.03)	5
4.6	4.68 (0.77)	5

### Comparative and Correlative Analysis

#### ISB Differences Based on User’s Type

**Figure 2:**
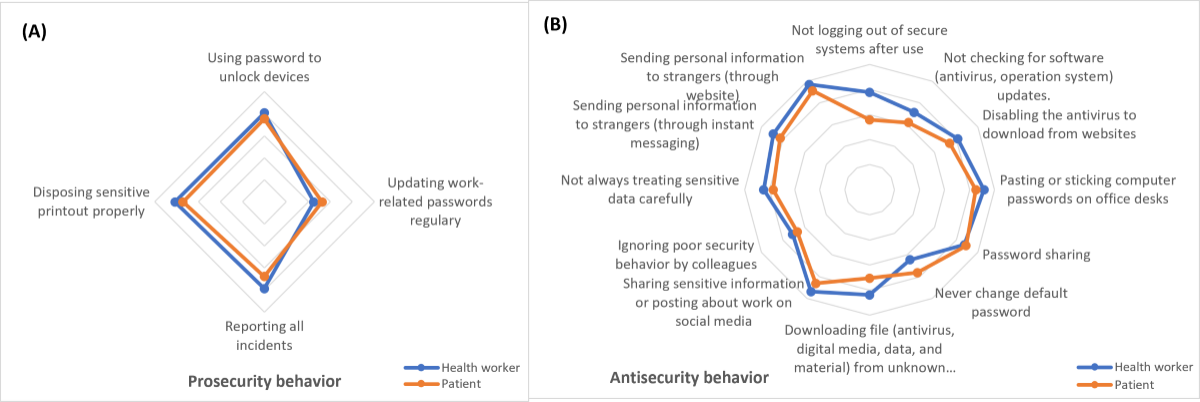
(A) Prosecurity behavior (B) and antisecurity behavior differences based on the user’s type.

There are 16 behaviors significantly different between health worker and patient, consisting of 4 prosecurity (Figure 2A) and 12 antisecurity (Figure 2B) behaviors. The health worker has better ISB than the patient since having a higher mean value in almost all different behaviors. However, patients have better behavior regarding password management than health workers. Patients more frequently update passwords, change default passwords, and rarely share passwords than health workers.

#### ISB Differences Based on Gender

**Figure 3:**
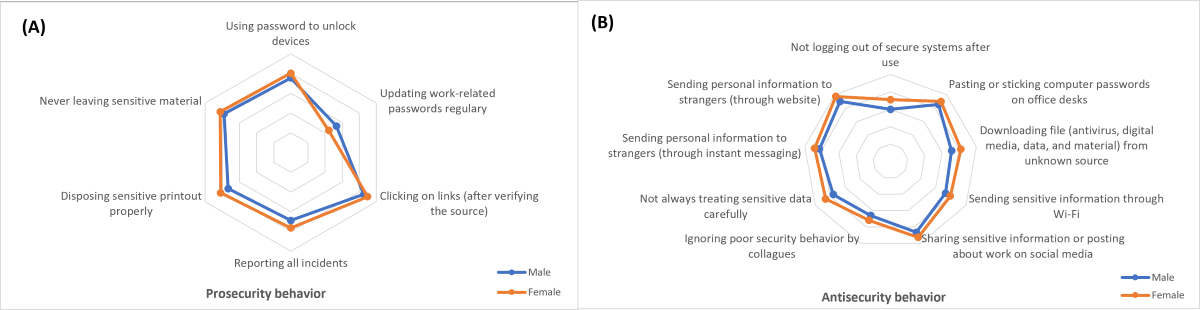
(A) Prosecurity behavior (B) and antisecurity behavior differences based on gender.

Figure 3 shows 15 security behaviors that differ between male and female users, consisting of 6 prosecurity (Figure 3A) and 9 antisecurity (Figure 3B) behaviors. Female users have better ISB than males in practicing security protection and avoiding risky behavior. Male users are only better at updating work-related passwords regularly.

#### ISB Differences Based on Age Group

**Figure 4:**
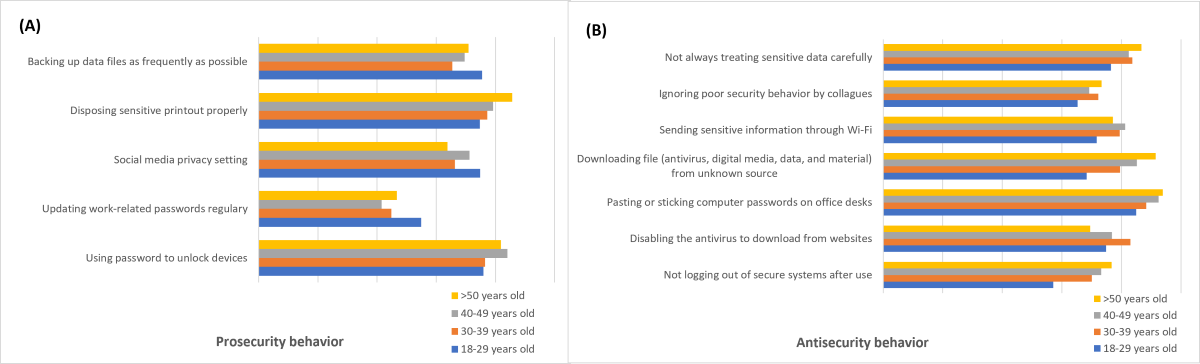
(A) Prosecurity behavior (B) and antisecurity behavior difference based on age group.

According to the age group, there are 12 security behavior differences, consisting of 5 prosecurity (Figure 4A) and 7 antisecurity (Figure 4B) behaviors. Younger users (18-29 years old) are better at security protection than other groups, especially in backing up data files, changing social media privacy settings, and regularly updating work-related passwords. There is no specific age group that exhibits less frequent prosecurity behavior. Meanwhile, older users (>50 years old) are better at practicing less antisecurity behavior, but younger users (18-29 years old) more often practice antisecurity behavior.

#### ISB Differences Based on the Education Level

**Figure 5:**
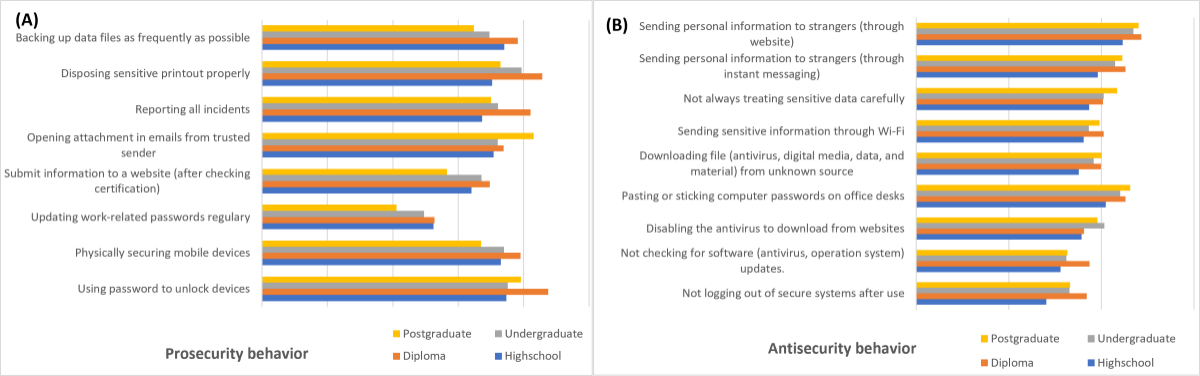
(A) Prosecurity behavior and (B) antisecurity behavior differences based on the education level.

A total of 17 behavior indicators are different based on education level, which are 8 prosecurity (Figure 5A) and 9 antisecurity behaviors (Figure 5B). Diploma users have better security behavior, are more frequently prosecurity, and have less antisecurity behavior than other groups. Postgraduate users are less likely to engage in prosecurity behavior except for opening an email attachment from a trusted sender. Meanwhile, the high school users’ group is most often practicing antisecurity behavior.

### ISB Correlation to Age and Education Level

This study included further comparative and correlative analyses. A correlational analysis was conducted to determine the relevance of the association between ISB and age and level of education, which is ordinal data. While the mean value of each group descriptively allows for comparison research to assess the relevance of the association between security behavior and gender and user type. The correlative test uses the Spearman correlation coefficient, with a significance value of (2-tailed) *P*=.05 indicating a significant association between ISB indicators and HIS users’ age or educational level.

Table 6 displays the correlation research results for prosecurity behavior with age and educational level. The behavior of users to use passwords to unlock devices used to access HIS (1.2) and ensure sensitive printouts (such as medical resumes, test results, and prescriptions) are appropriately destroyed before disposal (4.1) is significantly positively connected with age. This demonstrates that older HIS users are more likely to engage in both activities. Meanwhile, the behavior of HIS users to update HIS passwords on a regular basis (2.3), maintain social media privacy (3.4), and back up essential data on HIS (4.3) shows a negative association with age, with older users doing it less frequently. The correlation of HIS user behavior for not opening email attachments from unknown senders (3.3) shows a positive correlation with education level, with the higher the educational level of HIS users, the more frequently this behavior occurs. Because of the negative link, HIS users with higher levels of education change HIS account passwords (2.3) less frequently and back up essential data on HIS (4.3).

Table 7 shows the correlative study results for antisecurity behavior with age and education. The behavior of not logging out of HIS (1.4), sticking passwords in the open (2.4), downloading files from unofficial sources (3.6), sending sensitive information using public Wi-Fi (3.7), sharing sensitive information on social media (3.8), doing nothing when colleagues commit security breaches (3.10), not treating sensitive data carefully (4.4), and sending personal information to unknown websites (4.6) significantly positively correlated with age, so that the older HIS users tend to do less of this behavior. While the educational level of HIS users has a positive correlation with the behavior of disabling antivirus (1.6), pasting passwords (2.4), downloading files from unofficial sources (3.6), not treating sensitive data with care (4.4), and sending sensitive data through instant messaging without verifying the authenticity of the account (4.5). This shows that the higher the educational level of HIS users, the less likely they are to engage in this behavior.

**Table 6:** Correlative test results for prosecurity behaviors.

Item code	Age		Education	
	Correlation coefficient	*P* value	Correlation coefficient	*P* value
1.1	0.037	.45	0.037	.45
1.2	0.127	.009	0.009	.86
1.3	–0.023	.64	–0.078	.11
2.1	0.015	.76	–0.043	.38
2.2	0.046	.35	–0.031	.53
2.3	–0.191	<.001	–0.134	.006
3.1	–0.047	.33	–0.073	.14
3.2	0.058	.23	0.015	.76
3.3	0.064	.19	0.109	.03
3.4	–0.163	.001	–0.092	.06
3.9	0.060	.22	0.006	.90
4.1	0.131	.007	0.014	.78
4.2	0.045	.36	0.015	.76
4.3	–0.104	.03	–0.133	.006

**Table 7:** Correlative test results for antisecurity behaviors.

Item code	Age		Education	
	Correlation coefficient	*P* value	Correlation coefficient	*P* value
1.4	0.257	<.001	0.086	.08
1.5	0.089	.07	–0.004	.94
1.6	0.028	.57	0.116	.02
2.4	0.155	.001	0.112	.02
2.5	0.038	.44	–0.013	.79
2.6	–0.022	.65	0.089	.07
3.6	0.368	<.001	0.099	.04
3.7	0.160	.001	0.026	.60
3.8	0.118	.02	0.064	.19
3.10	0.136	.005	0.006	.90
4.4	0.169	.001	0.121	.01
4.5	0.094	.06	0.115	.02
4.6	0.112	.02	0.075	.13

## Discussion

### Principal Results

There is no previous study comparing the security behaviors of health workers and patients directly. This study shows that patients update and change default passwords more frequently. Patients also share their HIS password less often than health workers, who use HIS to work and share information with their colleagues. In contrast with a previous study [22], mHealth end users do not change their password regularly but use strong and different passwords. Health workers are more likely to use the same password due to stress factors in the health care environment [19]. This study also shows that health workers use the default password, which is usually the same for all accounts. They can easily ask their colleagues if they forget the password. The high workload sometimes causes health workers not to be able to access the HIS on their own, so they ask a coworker to help them by using their passwords. There is a significant difference in prosecurity behavior between health care workers and patients when it comes to reporting security events or disruptions to the HIS manager. Most of the staff will report incidents, but most of the patients will not. As a result, if there is a HIS problem, HIS managers must engage more with patients or provide more information about complaint services. User-reported events can be used to detect attacks or larger issues. Patients frequently do not log out after using HIS, which makes a significant impact in antisecurity behavior. This might happen since patients use their own devices to access the HIS, so they can feel safer if they do not log out, but health workers use institutional devices that someone else can use.

This study shows female and male users’ ISBs are different as a whole or in several indicators. Women tend to adopt prosecurity practices and avoid antisecurity behaviors, except for updating HIS password regularly. This result contradicts previous studies that took health workers [25] and nonhealth workers [32,33] as research participants and revealed that males are more likely to adhere to security controls and avoid risky behavior. However, a previous study [34] that included health workers as part of its research revealed that females are more likely to comply with security policies. There is a significant disparity in prosecurity behavior between both genders, with male users changing their HIS passwords more regularly and female users being more cautious while deleting sensitive information such as medicine prescriptions, ultrasound printouts, or medical resumes. Antisecurity behavior that differs substantially between male and female users includes not closing the system after usage, downloading files from unknown sources, and not frequently managing sensitive data with care. Female users are less likely to engage in all 3 of these behaviors. The causes for the disparities in behavior between male and female users must be investigated further.

In general, the older the HIS user, the more frequently they engage in prosecurity behavior and seldom engage in antisecurity behavior. However, younger HIS users are more likely to change passwords on a regular basis, set privacy on social media, and back up crucial data. It is supported by previous study where younger users are more likely to use security controls on mobile devices [25], but older users generally are more cautious with sensitive information [26]. The younger HIS users are more likely to embrace detailed processes. Still, most older people groups evaluate and accept informal security measures as a more seasoned response to security problems [24]. Older users (>40 years old) have better overall behavior. This is demonstrated by a correlation analysis between user age and security behavior, particularly antisecurity behavior, where the older the user, the less likely it is to engage in antisecurity behavior. However, the younger the user, the more frequently they change passwords and social media privacy settings.

This finding shows that education level mostly influences the differences in prosecurity behavior. However, higher education does not guarantee better ISB for HIS users. It is supported by a previous study [24] that revealed security policy compliance was lower at the master’s level than at the undergraduate level. Overall, users with a diploma education behave better than other age groups. However, these disparities in behavior vary sufficiently so that, according to the correlative analysis, there isn’t a very substantial association between education level and user security behavior. Furthermore, this study indicates that the higher the education of HIS users, the less frequently they change passwords and back up essential data.

Overall, the frequency with which HIS users engage in risky security behavior is lower than the frequency with which behavior is predicted. Because patients have a lower degree of security behavior than health care workers, information security education for HIS users is critical. This study adds a deeper understanding of HIS user behavior by age, gender, and educational level. Health care facilities management may personalize education programs and formats to the target group of end users.

### Implication and Limitations

These findings suggest the necessity for security education programs specific to user demographics and the categories of security behaviors the organization wants to enhance. Differences primarily influence antisecurity behavior in user roles, age, and gender, but education level affects both equally. This study demonstrates distinctions between prosecurity and antisecurity behaviors that have not been thoroughly examined in earlier studies. Health care facilities should focus on user-specific needs, knowledge, ability, and work limitations to reduce conflict between protection and work efficiency goals and develop customized security training programs [21].

Security education for the patient should be considered since they do antisecurity more often than health workers and can pose security threats. This study also differs from most previous studies because female users exhibit more secure behavior than males. Younger HIS users are more susceptible to engaging in risky security behavior, but they are also more likely to engage in prosecurity behavior, while older users are better at preventing antisecurity behavior. Based on educational degree, there are significant disparities in ISB. Users with a high school education are more susceptible to antisecurity behavior, thus they should be educated on information security risks. As users with undergraduate and postgraduate degrees are less likely to engage in prosecurity behavior, technical security training is required. Some behaviors that can be improved include regularly updating the HIS password, setting the device screen to automatically lock when not in use, not logging out after using the HIS, verifying website security before sending sensitive information, and delaying software updates such as operating system and antivirus updates. Security threats posed by HIS user behavior can be mitigated by including technological security measures into HIS, such as an automated logout function on HIS and user devices after a given amount of inactivity and implementing a password validity period for a set length of time. HIS users should be educated on potential risks that might occur if the software used is not kept up to date, as well as how to assess the security of a website.

This study uses a self-reported survey and does not distinguish the types of health facilities. It might cause bias because the security behavior described by the respondents’ answers can apply to a particular health facility. Additionally, this work does not thoroughly investigate the relationship between ISB and HIS features and the used devices. For instance, patients use smartphones more frequently to register through mobile apps, while health workers use desktop computers more frequently to access electronic medical records. Current technology adopting artificial intelligence might bring new security risks to health care systems [35]. Future research can investigate more relevant information security knowledge and behavior to cope with security risks from those technologies. This study assesses the ISB of HIS users based on a history of security events in health institutions and potential security threats. The influence of this user behavior on information security in associated health institutions has not been studied further. Future research can do a longitudinal study on a specific health institution to see the impact of this behavior and make measurements before and after introducing suitable security measures.

### Conclusions

Health care providers other than hospitals are still understudied. Studies related to both prosecurity and antisecurity behaviors show that the factors preventing protection can be different from the factors promoting information security violations. Therefore, both types of security behavior are necessary to be investigated for further research. The development of technological solutions used by health facilities since the COVID-19 outbreak, such as telemedicine and mHealth apps, has caused the coverage of HIS users to expand. Protection of health information security does not only rely on health care professionals but also on patients who participate in managing their own personal data. Information security risk does not only come from internal users at the health care provider but also from external users who have access rights to the system. Therefore, studies on ISB in the context of health organizations need to understand the patient’s perspective, which is still rarely studied.

## Abbreviations

CCSB: counterproductive computer security behaviors
HAIS-Q: Human Aspects of Information Security Questionnaire
HIS: health information system
ISB: information security behavior
mHealth: mobile health
RQ: research question
RScB: Risky Cybersecurity Behaviors Scale
SeBIS: Security Behavior Intentions Scale

## Data Availability

The data sets generated and analyzed during this study are available from the corresponding author on reasonable request.
